# Gene-Metabolite Expression in Blood Can Discriminate Allergen-Induced Isolated Early from Dual Asthmatic Responses

**DOI:** 10.1371/journal.pone.0067907

**Published:** 2013-07-02

**Authors:** Amrit Singh, Masatsugu Yamamoto, Sarah H. Y. Kam, Jian Ruan, Gail M. Gauvreau, Paul M. O'Byrne, J. Mark FitzGerald, Robert Schellenberg, Louis-Philippe Boulet, Gabriella Wojewodka, Cynthia Kanagaratham, Juan B. De Sanctis, Danuta Radzioch, Scott J. Tebbutt

**Affiliations:** 1 James Hogg Research Centre, St. Paul’s Hospital, University of British Columbia, Vancouver, British Columbia, Canada; 2 Institute for HEART+LUNG Health, Vancouver, British Columbia, Canada; 3 NCE CECR PROOF Centre of Excellence, Vancouver, British Columbia, Canada; 4 Department of Medicine, McMaster University, Hamilton, Ontario, Canada; 5 Division of Respiratory Medicine, Department of Medicine, University of British Columbia, Vancouver, British Columbia, Canada; 6 Vancouver Coastal Health Research Institute, Vancouver General Hospital, Vancouver, British Columbia, Canada; 7 Centre de Pneumologie de L’Hopital, Université Laval, Sainte-Foy, Quebec, Canada; 8 Department of Human Genetics, McGill University, Montreal, Quebec, Canada; 9 Central University of Venezuela, Institute of Immunology, Caracas, Venezuela; Harvard Medical School, United States of America

## Abstract

Some asthmatic individuals undergoing allergen inhalation challenge develop an isolated early response whereas others develop a dual response (early plus late response). In the present study we have used transcriptomics (microarrays) and metabolomics (mass spectrometry) of peripheral blood to identify molecular patterns that can discriminate allergen-induced isolated early from dual asthmatic responses. Peripheral blood was obtained prior to (pre-) and 2 hours post allergen inhalation challenge from 33 study participants. In an initial cohort of 14 participants, complete blood counts indicated significant differences in neutrophil and lymphocyte counts at pre-challenge between early and dual responders. At post-challenge, significant genes (*ALOX15*, *FADS2* and *LPCAT2*) and metabolites (lysolipids) were enriched in lipid metabolism pathways. Enzymes encoding for these genes are involved in membrane biogenesis and metabolism of fatty acids into pro-inflammatory and anti-inflammatory mediators. Correlation analysis indicated a strong negative correlation between *ALOX15*, *FADS2*, and *IL5RA* expression with 2-arachidonoylglycerophosphocholine levels in dual responders. However, measuring arachidonic acid and docosahexaenoic acid levels in a validation cohort of 19 participants indicated that the free form of DHA (nmoles/µg of protein) was significantly (p = 0.03) different between early and dual responders after allergen challenge. Collectively these results may suggest an imbalance in lipid metabolism which dictates pro- (anti-) inflammatory and pro-resolving mechanisms. Future studies with larger sample sizes may reveal novel mechanisms and therapeutic targets of the late phase asthmatic response.

## Introduction

Asthma is the most common chronic lung disease, but remains poorly understood due to its complexity and heterogeneity [1]. Asthma is characterized by reversible narrowing of the airways, airway inflammation and airway remodeling [2]–[5]. Current physiological or functional tests such as clinical symptoms or lung function tests have not been shown to reflect airway inflammation and clinical outcome [6].

The allergen inhalation challenge is a useful clinical model in order to study the mechanisms underlying asthmatic responses [7]. The early asthmatic response (EAR) is initiated upon allergen inhalation and results in the activation of IgE-bearing cells such as mast cells and basophils [8]–[10]. Upon degranulation, these cells release proinflammatory mediators such as histamine and eicosanoids triggering bronchoconstriction and increased vascular permeability [11], [12]. The late asthmatic response (LAR) occurs 4 to 6 hours [13] after allergen exposure and is characterized by cellular infiltration of the airway, increased bronchovascular permeability, and mucus secretion [14]. Fifty to 60% of allergic asthmatic individuals develop both an EAR and LAR (dual responders; DRs) following allergen inhalation challenge, whereas 30 to 40% of allergic asthmatic individuals develop an isolated early response (early responders; ERs) after allergen challenge [7]. The molecular mechanisms leading to the early and late asthmatic responses are not fully understood.

Recent advancements in ‘omics’ technologies such as transcriptomics and metabolomics have enabled the study of complex diseases such as asthma [15], [16]. However, the success of these technologies is limited by confounding factors such as the heterogeneity of study populations, and the effect of medications on gene expression [17], [18]. Therefore, careful selection of subjects and clinically relevant samples should enable greater understanding of the underlying mechanisms and reveal novel therapeutic targets for the treatment of allergic asthma.

Our laboratory has previously shown that significant changes in the whole blood transcriptome (mRNA and miRNA) of mild atopic asthmatic individuals can be identified two hours after allergen inhalation challenge [19], [20]. Previous studies comparing ERs and DRs undergoing allergen inhalation challenge have investigated changes in inflammatory progenitor cells in peripheral blood [21] and sputum [22]. In addition, the change in IL-10 producing CD4^+^ cells after allergen inhalation challenge has been shown to differ between ERs and DRs [23]. In addition, we have recently reported significant differences in the plasma proteome between ERs and DRs [24]. In the present study, we demonstrate the utility of applying omics-based approaches to peripheral blood in order to identify molecular patterns that can discriminate allergen-induced isolated early from dual asthmatic responses.

## Methods

### Asthma Cohorts

This study was approved by the Institutional Review Boards of the participating health research institutes (McMaster University, Université Laval, and University of British Columbia). Following written informed consent, 33 individuals participated in the allergen inhalation challenge as part of the AllerGen Clinical Investigator Collaborative (CIC). All study participants had predicted FEV_1_ (forced expiratory volume in one second) [25] greater than 70% and baseline methacholine PC_20_ (the provocative concentration of methacholine causing a 20% fall in FEV_1_) less than 16 mg/ml [26]. The study participants were non-smokers with stable, mild atopic asthma, free of other lung disease. Exclusion criteria included: use of inhaled corticosteroids and use of other asthma mediation with the exception of infrequently used β_2_-agonists which were withheld 8 hours prior to spirometry measurements [27]. All study participants were selected due to their hypersensitivity to various allergens using skin prick tests. The discovery cohort consisted of 14 participants whereas an additional 19 participants were recruited for the validation phase.

### Methacholine Challenge Test and Allergen Inhalation Challenge

A methacholine challenge test (MCT) was conducted one day prior and one day following allergen inhalation challenge. On day one, each participant underwent a MCT where methacholine [PC_20_] was calculated using the Cockcroft equation [26]. A post MCT was conducted 24 hours after the allergen inhalation challenge in order to detect an allergen induced shift in airway hyperresponsiveness. The allergen inhalation challenge was conducted on the second day, where spirometry measurements were assessed at regular intervals for up to 7 hours, inclusive. Skin prick tests were used to determine the dose of allergen extract for inhalation. The allergen dosage was administered in a doubling dosage until a drop in FEV_1_ of 20% was achieved in all participants. Study participants were classified as early responders (ERs) if the initial drop in FEV_1_ resolved back to baseline within 1 to 3 hours of allergen inhalation and if the maximum drop in FEV_1_ between 3 to 7 hours was less than 15%. Study participants were classified as dual responders (DRs) if in addition to the early response, the participants experienced a maximum drop in FEV_1_ of 15% or greater between 3 to 7 hours of allergen inhalation. Participants with a maximum late phase drop less than 15% but required a lower dose of methacholine [PC_20_] on the second MCT compared to the first MCT were also classified as DRs.

### Blood Collection and Processing

A standard operating protocol exists for all blood collection and processing at participating AllerGen CIC sites. Ten milliliters of venous blood was obtained from each subject, prior to challenge (pre) and 2 hours post-challenge. Leukocyte-enriched (PAXgene Blood RNA tubes, PreAnalytiX, Qiagen/BD, Valencia, CA, USA) and plasma fractions (in multiple aliquots) were freshly prepared from the blood and frozen at −80°C, after which RNA was extracted using standard Qiagen kits. Complete blood counts and differentials (CBCs/differentials) were obtained at the time of each blood draw (via a 3 ml EDTA blood tube, drawn immediately prior to the PAXgene Blood RNA tube sample). CBCs/differentials were obtained for all study participants in the discovery cohort.

### Transcriptomics

Whole transcript gene expression analysis of pre and post RNA samples (28 samples) of the discovery cohort was performed using Affymetrix Human Gene 1.0 ST Arrays at the Centre for Translational and Applied Genomics (CTAG) in Vancouver, British Columbia. All microarray data (CEL) files have been deposited in NCBI’s Gene Expression Omnibus (GEO Series GSE40240). The ‘affy’ package [28] was used to import CEL files, and annotate Affymetrix IDs to gene symbols. The farms package (version 1.6.0) was used for preprocessing and filtering of non-informative probe sets [29], [30]. Preprocessing included the Robust MultiArray Average (RMA) background correction, quantile normalization, and Factor Analysis for Robust Microarray Summarization (FARMS). The summarization step makes use of factor analysis to determine a common factor (z) in the set of probes measuring the same target gene (probe set). In the process, parameters representing the signal and noise for each probe set are calculated. Informative/Non-informative (I/NI) calls use these parameters in order to filter out probe sets with little correlation amongst their probes (non-informative probe sets). The use of I/NI-calls for filtering of probe sets avoids having to set arbitrary thresholds such as for overall variance and mean cut-offs which may eventually affect downstream analysis [31]. All software packages used to preprocess the microarray data can be accessed through Bioconductor (http://www.bioconductor.org/). All analyses were performed in the statistical computing program R 2.14.2 [32].

### Metabolomics Using the Metabolon Platform

Metabolite profiling as well as informatics were performed by Metabolon Inc. (Durham, North Carolina, United States). Samples from the discovery cohort were extracted and prepared for analysis using Metabolon’s standard solvent extraction method. The extracted samples were divided into equal parts for analysis on the GC-MS and LC-MS/MS platforms. Also included were several technical replicate samples created from a homogeneous pool, containing a small amount of all study samples. The Metabolon informatics system consisted of four major components, the Laboratory Information Management System (LIMS), the data extraction and peak-identification software, data processing tools for quality control and compound identification, and a collection of information interpretation and visualization tools. The hardware and software foundations for these informatics components were the LAN backbone, and a database server running Oracle 10.2.0.1 Enterprise Edition. General platform methods are described in the Supplementary Information.

### Lipid Profiling

Arachidonic acid (AA) and docosahexaenoic acid (DHA) were profiled using plasma samples in a validation cohort of 19 study participants. Briefly, plasma was first suspended in 1 mM butylated hydroxyanisole (BHA) in chloroform and methanol (2∶1) in order to protect the integrity of the samples. Following a previously described method [33], lipids were extracted from these plasma samples using 1.9 ml of chloroform/methanol (2∶1 v/v) and 1 ml of cold water. From the organic phase, aliquots were used for phospholipid analysis by thin-layer chromatography (TLC) [34]. Lipids were separated from samples by TLC and detected by iodine. After scraping from the plate, the fatty acids were esterified using diazomethane and the esters were quantified using gas chromatography/mass spectrometry (Hewlett Packard5880A, WCOT capillary column (Supelco-10, 35 m×0.5 mm, 1 µm thick)) using commercial standards (Sigma-Aldrich, Oakville, ON, Canada). The amount of lipids per sample was normalized to the protein concentration measured by the bicinchoninic assay (BCA).

### Statistical Analyses

Moderated robust regression in the Linear Models for MicroArrays (limma) package [35] was used in order to determine differential changes in gene expression between early and dual responders. Limma moderates the gene-wise residual variance by combining it with the global mean residual variance across all genes, in order to control for the number of false positive discoveries. A Benjamini Hochberg false discovery rate (FDR) [36] of 10% was used for all limma analyses. Robust regression [37] in the MASS package was used to determine differentially expressed metabolites and lipids using a p-value cut-off of 0.05. Robust test statistics were used for all analyses in order to attenuate the effects of outliers in small sample sizes. In addition, in order to avoid over fitting each variable of interest, ERs vs. DRs at pre-challenge, and at post-challenge (scaled to pre-challenge; post-challenge divided by pre-challenge levels) were considered in separate linear models. Age and sex have been adjusted for in all comparisons (discovery and validation). Regularized Canonical Correlation Analysis (RCCA), an extension of CCA [38], highlights the correlation between two data sets where the number of variables (genes/metabolites) is much larger than the sample size (see Section 3 in [39]). RCCA in the mixOmics package was used in order to identify highly correlated clusters of differentially expressed genes and metabolites in ERs and DRs, respectively. All software packages used to perform the statistical analyses can be accessed through Bioconductor (http://www.bioconductor.org/) and The Comprehensive R Archive Network (CRAN) (http://cran.r-project.org/).

### Gene-set Enrichment Analysis

Top biological functions and canonical pathways of differentially expressed genes were analyzed using Ingenuity Pathway Analysis (IPA). The filtering criteria included; organ: lung, and cells: immune cells. Gene networks consisting of hypothetical and proven relationships were also generated using IPA. Fisher’s Exact Test p-value of 0.05 and Benjamini Hochberg adjusted p-value (FDR) of 0.05 were used to determine significant canonical pathways and biological pathways.

## Results

### Asthma Cohort

**Figure 1:**
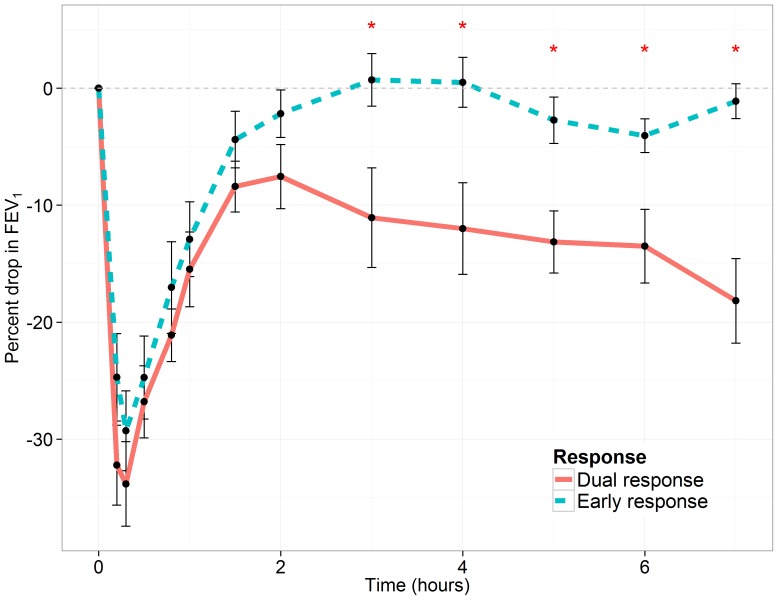
Lung function during allergen inhalation challenge. Forced expiratory volume in 1 second (FEV_1_) measurements at 0.2 h, 0.3 h, 0.5 h, 0.8 h, 1 h, 1.5 h, 2 h, 3 h, 4 h, 5 h, 6 h and 7 h for eight early and six dual responders. FEV_1_ measurements are statistically different (*p<0.05) between early and dual responders at each of the time points between three and seven hours inclusive. The p-value for each time point comparing ERs and DRs was computed using a robust linear model (see Methods).

Table 1 displays the demographics for the initial 14 study participants (eight ERs and six DRs), that participated in the cat allergen inhalation challenge. Both groups were age-matched and met the initial percent drop in forced expiratory volume in one second (FEV_1_) criteria; a drop of greater than 20% (Figure 1). During the late phase (between 3 to 7 hours), the maximum percent drop in FEV_1_ in the DRs (21.3±3.2%) was approximately 4 times greater than that in ERs (5.1±1.4%).

**Table 1:** Demographics of study participants of the discovery cohort.

Patient ID	Age (year)	Sex (M:F)	Pre [PC_20_] (mg/mL)	Post [PC_20_] (mg/mL)	Allergen Induced Shift^b^	% Fall in FEV1	
						Early	Late
**ER**							
1	28	F	12.8	ND	ND	−20.3	−4.8
2	34	F	2.8	6.1	0.4	−21	−1.5
3	27	M	4.5	1.8	2.5	−34.4	0
4	42	F	5.3	8.6	0.6	−42.1	−11.1
5	29	F	0.4	ND	ND	−44.3	0
6	31	M	11.8	16	0.7	−24.2	−7.5
7	28	F	9.4	16	0.6	−27.1	−7.1
8	42	M	0.1	ND	ND	−23	−9
Mean ± SE	32.6±2.2	3∶5	2.8^a^	7.5^a^	1.0±0.4	−29.6±3.2	−5.1±1.4
**DR**							
9	23	F	0.3	0.2	1.5	−38.9	−31.8
10	26	F	5.1	1.5	3.4	−31.4	−14.9
11	49	F	3.6	1.0	3.6	−25.3	−12.6
12	26	M	0.9	1.0	0.9	−31.5	−15.6
13	27	F	0.6	0.1	6	−48.3	−25.8
14	52	F	ND	ND	ND	−33	−27
Mean ± SE	33.8±3.3	1∶5	1.3^a^	0.5^a^	3.0±0.8	−34.7±3.2	−21.3±3.2^c^

ND - Not determined; all study participants were challenged with cat allergen.

a : geometric mean (PC_20_ values are measured on a log scale);

b : [PC_20_]_pre_/[PC_20_]_post._

c p<0.05 versus ER group.

### Blood Differential Counts

**Figure 2:**
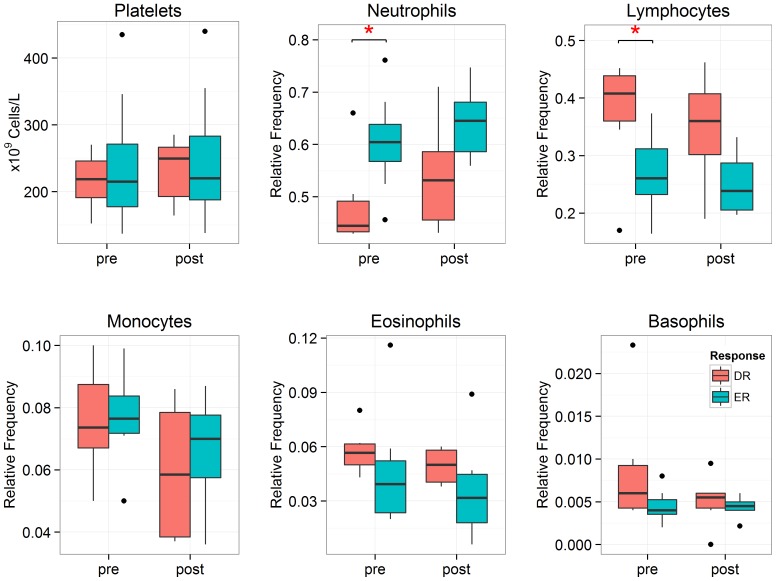
Complete blood counts. Relative frequencies of various leukocytes obtained at pre and post-challenge. The relative neutrophil counts were significantly (p = 0.01) elevated in ERs compared to DRs, whereas the relative lymphocyte counts were significantly reduced (p = 0.02) in ERs compared to DRs at pre-challenge. The p-value was computed using a robust linear model (see Methods); * denotes p<0.05.

Complete blood counts with differentials were obtained from all samples in the discovery cohort. At pre-challenge, absolute neutrophil counts were significantly (p = 0.04) greater in ERs (3.5±0.4×10^9^ cells) compared to DRs (2.6±0.5×10^9^ cells). Figure 2 indicates that at pre-challenge, the relative levels of neutrophils were significantly (p = 0.01) greater in ERs (60±3.0%) compared to DRs (50±4%), whereas relative levels of lymphocytes were significantly (p = 0.02) reduced in ERs (30±2%) compared to DRs (40±4%). No significant differences in cell counts were identified post-challenge. All differential measures are presented in Table S1.

### Profiling Gene Expression and Metabolite Levels

32,321 probe sets on the Human Gene ST 1.0 microarray were used to profile blood leukocyte gene expression. Pre-filtering for informative probe sets (see Methods) resulted in 770 probe sets of which 497 annotated probe sets were retained for further analysis. From the same samples, the plasma was analyzed for 292 named and 201 unnamed metabolites; only the named metabolites were kept for downstream analyses.

### Differentially Expressed Genes and Metabolites at Pre-challenge

At pre-challenge, 72 (11 over and 61 under-expressed) differentially expressed probe sets in DRs relative to ERs were identified at an FDR of 10%, after adjusting for age and sex. Ingenuity Pathway Analysis (IPA) indicated Connective Tissue Disorders, Immunological Disease, Inflammatory Disease and Skeletal and Muscular Disorder as the top biological functions at an FDR of 5% (Figure S1). Significant genes, such as Interleukin 8 receptor, alpha (*CXCR1*), Nicotinamide phosphoribosyltransferase (*NAMPT*), C-C chemokine receptor type 7 (*CCR7*) and Lymphoid enhancer-binding factor 1 (*LEF1*) are abundantly expressed by neutrophils and lymphocytes. In fact the differences in gene expression between ERs and DRs are consistent with the differences in cell-type frequencies between ERs and DRs. (Figure 2). Therefore it is likely that the differential gene expression is confounded by the white blood cell count differences between ERs and DRs at pre-challenge rather than true differences in cellular gene expression.

Eight metabolites were found differentially expressed (p<0.05) between ERs and DRs at pre-challenge. Plasma levels of six metabolites including bilirubin, 4-vinyl phenol sulphate, 2-arachidonoylglycerophosphocholine, methionine, N-acetylglycine and malate were significantly elevated in DRs compared to ERs. Methyl palmitate and mannose were significantly under-expressed in DRs relative to ERs.

### Differentially Expressed Genes and Metabolites at Post-challenge

**Figure 3:**
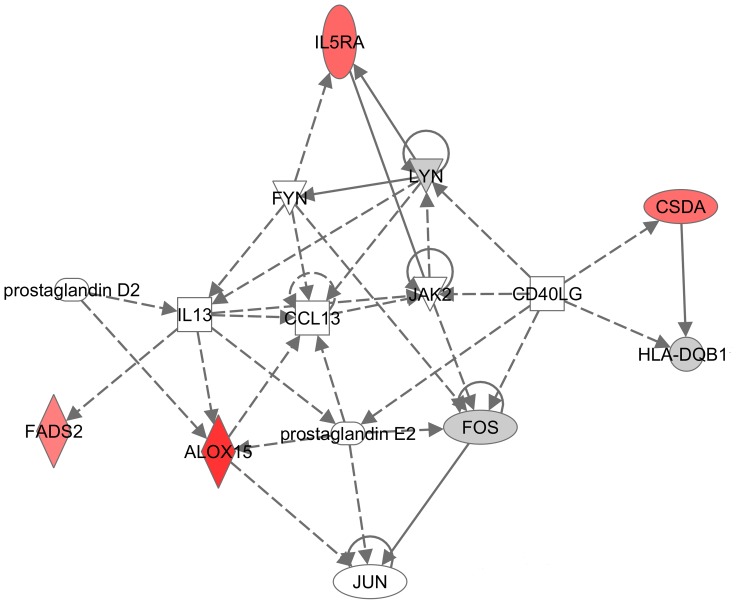
Gene network. Ingenuity Pathway Analysis network of differentially expressed genes at post-challenge comparing ERs and DRs. Dash lines indicate indirect relationships, whereas solid lines indicate direct relationships. The red colour indicates significant genes at an FDR of 10%. The grey colour indicates non-significant genes at an FDR of 10%.

25 probe sets were differentially expressed post-challenge at an FDR of 10%, after adjusting for age and sex (Table S2A). Six of these probe sets were up-regulated whereas 4 probe sets were down-regulated in both ERs and DRs after challenge. The gene encoding RPL9 (ribosomal protein L9) was up-regulated in ERs and down-regulated in DRs at post-challenge. The remaining 14 genes were down-regulated in ERs and up-regulated in DRs after challenge. IPA revealed Lipid Metabolism (FDR<5%) and Linoleic Acid Metabolism (p<0.05) as the top biological function and canonical pathway, respectively (Figure S2). Figure 3 shows a network of the differentially expressed genes consisting of the Interleukin 5 receptor alpha (*IL5RA*), Fatty acid desaturase 2 (*FADS2*), Arachidonate 15-lipoxygenase (*ALOX15*), and Cold shock domain protein A (CSDA). FADS2 (Δ6-fatty acid desaturase) is a key enzyme in the biosynthesis of ω-6 and ω-3 fatty acids such as arachidonic acid (AA) and docosahexaenoic acid (DHA) [40]. ALOX15 (15-LOX-1) can metabolize AA into lipoxins which have anti-inflammatory properties [41]. 15-LOX-1 can also produce pro-inflammatory mediators such as eoxins by metabolizing arachidonic acid in eosinophils and mast cells [42]. In addition, 15-LOX-1 can also metabolize DHA into 17-series DHA metabolites such as resolvins and protectins which have anti-inflammatory and pro-resolving properties and are more potent than metabolites derived from AA [43]. Table S2A indicates that Lysoposphatidylcholine acyltransferase 2 (*LPCAT2*), which is involved in membrane biogenesis [44] was differentially expressed between ERs and DRs at post-challenge.

Eleven metabolites were differentially expressed (p<0.05) post-challenge (Table S2B). Four of these metabolites were up-regulated whereas 2 metabolites were down-regulated after challenge in both ERs and DRs. The metabolite 1-pentadecanoylglycerophosphocholine was up-regulated in ERs and down-regulated in DRs after challenge. There were 4 metabolites that decreased in ERs after challenge and increased in DRs after challenge. The list of 11 differentially expressed metabolites was mainly enriched with lipid and amino acid metabolism pathways. Lipids were either lysolipids such as 1-pentadecanoylglycerophosphocholine, 2-arachidonoylglycerophosphocholine, 1-linoleoylglycerophosphocholine or sterols/steroids such as andro steroid monosulfate 1, cortisol and 7-alpha-hydroxy-3-oxo-4-cholestenoate (7-Hoca). The amino acid metabolism pathway consisted of metabolites such as 4-hydroxyphenylacetate, 3-methyl-2-oxobutyrate and cysteine. Interestingly, the polypeptide bradykinin, hydroxy-pro (3) increased in DRs and decreased in ERs in response to allergen challenge.

**Figure 4:**
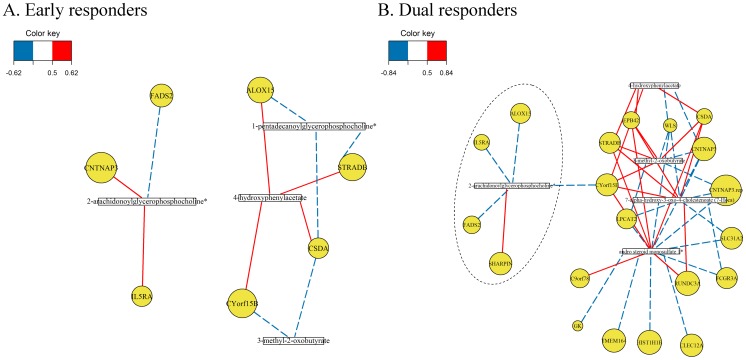
Network plots highlighting the correlation between ΔG and ΔM for early and dual responders. A. Gene-metabolite clusters for early responders (ΔG_25,8_ and ΔM_11,8_). B. Gene-metabolite clusters for dual responders (ΔG_25,6_ and ΔM_11,6_). A correlation coefficient cut-off of 0.5 is applied to both networks.

Regularized Canonical Correlation Analysis (RCCA) was used to highlight clusters of correlated genes and metabolites. The ratio between pre and post gene expression levels (post/pre) was calculated for the 25 and 11 differentially expressed genes and metabolites for 14 responders resulting in 2 datasets; ΔG_25,14_ (25 significant genes for 14 responders) and ΔM_11,14_ (11significant metabolites for 14 responders). Figure 4 displays the correlations between ΔG and ΔM independently for ERs (Figure 4A; ΔG_25,8_ and ΔM_11,8_) and DRs (Figure 4B; ΔG_25,6_ and ΔM_11,6_) using a correlation cut-off of 0.5. RCCA indicated greater numbers of highly correlated (r>0.5) gene-metabolite clusters in dual responders (Figure 4B) compared to early responders (Figure 4A). Interestingly in dual responders (Figure 4B; dotted circle), 2-arachidonoylglycerophosphocholine is able to link the key enzymes (*FADS2* and *ALOX15*) involved in lipid metabolism and *IL5R*A identified in Figure 3.

These results may suggest that, the interaction of lipid metabolizing enzymes with membrane phospholipids in white blood cells may differ between early and dual responders. However, validation of the underlying mechanism is beyond the scope of this study. Instead we looked to see how general mediators of inflammation which are also components of cellular membranes such as arachidonic acid (pro- and anti-inflammatory) and docosahexasnoic acid (anti-inflammatory and pro-resolving) respond to allergen inhalation challenge.

### ω-6 and ω-3 Polyunsaturated Fatty Acids

**Figure 5:**
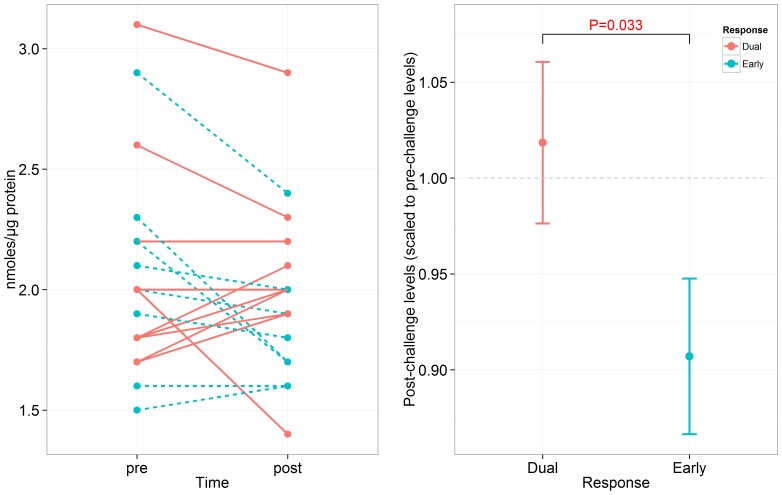
Levels of free docosahexaenoic acid in the plasma of early and dual responders undergoing allergen inhalation challenge. Levels of DHA (left) decrease in early responders from pre to post-challenge whereas no consistent change is observed in dual responders following allergen challenge. At post-challenge (scaled to pre-challenge levels), DHA was differentially expressed (p = 0.033) between ERs and DRs (right). The p-value was computed using a robust linear model (see Methods).

Both free and phospholipid forms of arachidonic acid (AA) and docosahexaenoic acid (DHA) were measured in a validation cohort of 19 (8 ERs and 11 DRs) study participants challenged with various allergens including cat, grass and ragweed (Table 2). The free fatty acid form is the fatty acid bound to albumin or on the surface of lipoproteins and usually refers to the metabolized lipid either from the hydrolysis of phospholipids or other complex lipids. The phospholipid (membrane bound) form is another structure which combines two fatty acids, usually at the *sn* position 2 in AA or DHA. Lipid levels were compared between ERs and DRs at post-challenge (scaled to pre-challenge) using a robust linear model adjusting for age and sex. At post-challenge, neither forms of AA were significant (Table S3), whereas only the free form of DHA was significant (p = 0.033) between ERs and DRs. Figure 5 shows that the free form of DHA decreases in ERs post-challenge compared to baseline levels whereas little change occurs in DRs.

**Table 2:** Demographics of study participants of the validation cohort.

	Age (year)	Sex (M:F)	Allergen	Pre [PC_20_] (mg/mL)	Post [PC_20_] (mg/mL)	Allergen Induced Shift^b^	% Fall in FEV1	
							Early	Late
**ER**								
1	25	M	Timothy Grass	0.08	ND	ND	−25.24	−11
2	36	F	Timothy Grass	0.64	ND	ND	−19.66	4.41
3	21	F	Ragweed	6.96	2.93	0.421	−33.1	2.11
4	33	M	Grass	13.3	21.1	1.587	−50	−2.5
5	21	F	Cat	0.59	ND	ND	−39.66	−1.72
6	30	F	Grass	1.81	ND	ND	−39.8	−11.7
7	21	F	Cat	9.6	ND	ND	−44.96	−12.4
8	43	M	Cat	1.94	ND	ND	−25.6	−10.8
Mean ± SE	28.8±2.9	3∶5		1.8^a^	7.9^a^	1.0±0.3	−34.8±3.8	−5.5±2.4
**DR**								
9	56	F	Cat	0.16	0.3	1.875	−23.22	−21.3
10	37	M	Timothy Grass	3.26	0.08	0.025	−60.61	−36.9
11	48	M	Orchard Grass	0.28	0.79	2.821	−25.16	−19.4
12	44	M	Cat hair	1.45	0.36	0.248	−41.33	−21.3
13	40	M	Grass	6.9	2.38	0.345	−21.21	−17.6
14	19	M	Grass	15.56	2.36	0.152	−42.99	−26.2
15	21	M	Ragweed	1.5	1.15	0.767	−55.73	−16.3
16	21	F	Cat	0.42	0.23	0.548	−44.06	−31.6
17	43	F	Cat	0.28	0.18	0.643	−26.92	−17.3
18	21	F	Cat	5.25	3.78	0.72	−47.22	−11.5
19	20	F	Grass	5.34	1.64	0.307	−23.61	−16.7
Mean ± SE	33.6±4.1	6∶5		1.5^a^	0.7^a^	0.8±0.3	−37.5±4.2	−21.5±2.2^d^

a geometric mean (PC20 values are measured on a log scale);

b [PC_20_]pre/[PC_20_]post;

c p<0.001 versus ER group.

## Discussion

Asthma is a complex disease consisting of multiple sub-phenotypes such that current therapies perform well in some phenotypes compared to others [45]. Therefore, well characterized study cohorts are required to identify new molecular targets of asthma. Despite the limited sample size of the present study, many potentially confounding factors have been accounted for through careful selection of study participants. The participants were selected from a homogenous group of non-smoking individuals with stable, mild, atopic asthma and free of other lung diseases. Detailed characterization of the participants was based on the development of allergen-induced bronchoconstriction, and airway responsiveness to methacholine. Although airway samples are commonly used to study the pathobiology of respiratory disease, such samples are obtained using invasive procedures. In the present study, we demonstrate the utility of applying ‘omics’ based approaches to peripheral blood in order to discriminate allergen-induced early from dual asthmatic responders (ERs from DRs).

Although many genes were differentially expressed between ERs and DRs prior to (pre-) allergen inhalation challenge, this may be influenced by significant differences in cellular frequencies between ERs and DRs (Figure 2). Figure 2 shows that the cellular frequencies of ERs and DRs move in parallel from pre to post-challenge, thus comparing post-challenge levels (scaled to pre-challenge levels, see Methods) was deemed less likely to be influenced by differences in cell-type proportions. Therefore greater focus was directed towards identifying significant molecular differences between ERs and DRs in response to the allergen inhalation challenge. These molecular targets may serve as effective tools for the diagnostic monitoring of asthmatic responses in clinical trials or as therapeutics targets for the attenuation of the late phase asthmatic response.

Differentially expressed genes at post-challenge were enriched for lipid metabolism and linoleic acid metabolism pathways (Figure S2). Figure 3 depicts genes within these pathways such as *FADS2* and *ALOX15* as well as *IL5RA*, which may suggest that these expression differences may be originating from eosinophils. Table S2A indicates that *EMR1*, an eosinophil-specific receptor was differentially expressed between ERs and DRs at post-challenge [46]. The mechanism of IL-5 on eosinophil survival involves the arachidonate pathway where phosphorylation of cytosolic phospholipase A_2_ α (cPLA_2_-α) induces up-regulation of arachidonic acid levels in eosinophils [47]. Allergen inhalation challenge stimulates these pathways in circulating blood eosinophils [47]. FADS2 is a key enzyme in the biogenesis of arachidonic acid and docosahexaenoic acid [48]. ALOX15 can produce pro-inflammatory mediators such as eoxins [42] and anti-inflammatory mediators such as lipoxins from arachidonic acid [49], as well as pro-resolving mediators such as resolvins and protectins from docosahexaenoic acid [41], [50]. Previous studies analyzing lipid mediators in bronchoalveolar lavage fluid have identified significant differences in ALOX15 metabolites between mild asthmatics and controls [42], [43]. The latter study indicated higher levels of ALOX15 metabolites in mild allergic asthmatics compared to healthy controls, and this difference increased following allergen challenge [43]. This is clear evidence that blood is a useful medium to depict lung inflammation since divergent oxylipin profiles can be observed in individuals with mild disease.

Lipid metabolism was also enriched in the list of significant metabolites at post-challenge. Cortisol, an immunosuppressive and anti-inflammatory molecule decreased in ERs and increased in DRs after challenge. Cortisol bound to carrier proteins in the blood can be released at the site of inflammation by the catalytic activity of neutrophil elastase [51]. This result may suggest that the uptake of plasma cortisol in ERs leads to reduced inflammation whereas this mechanism is impaired in DRs. Many lysolipids which are important components of cellular membranes were differentially regulated between ERs and DRs after challenge (Table S2B). The release and subsequent metabolism of these lipids into pro- or anti-inflammatory mediators may dictate the associated clinical response. Bradykinin, hydroxy-pro(3) was differentially expressed between ERs and DRs at post-challenge; increase in DRs and decrease in ERs from pre- to post-challenge. This result supports the well established role of bradykinin in asthma as a bronchoconstrictor and vasodilator [52]. We have previously shown that Kininogen (KN1), which is a precursor of bradykinin decreases in DRs and increases in ERs after challenge [24]. Therefore, plasma metabolomics reinforces the genomics analysis and identifies other biologically relevant molecules.

Although each data set (transcriptomics and metabolomics) is useful in identifying differences between ERs and DRs, considering each data set independently may result in some loss of information as both data sets were obtained from the same individuals. Current bioinformatics tools contain poor annotations for many of the differentially expressed metabolites; therefore integration with the transcriptomic data was approached using statistical means. Correlation analysis identified interesting patterns between differentially expressed genes and metabolites in ERs and DRs at post-challenge. Figure 4B shows that genes encoding for enzymes that can metabolize (*ALOX15*) or produce (*FADS2*) arachidonic acid were negatively correlated with the arachidonic acid lysolipid (2-arachidonoylglycerophosphocholine) in DRs after challenge. Although this network is biologically plausible, experimental work to validate the interactions amongst these molecules in peripheral blood is beyond the scope of this study.

Interestingly, in the validation cohort of 19 study participants (8 ERs and 11 DRs), docosahexaenoic acid instead of arachidonic acid was differentially expressed between ERs and DRs at post-challenge. The free form of DHA was down-regulated in ERs post challenge compared to pre-challenge whereas little change occurred in DRs from pre- to post-challenge (Figure 5). It may be possible that DHA is being taken up and metabolized in ERs leading to the resolution of inflammation [43], [50], [53] whereas no such effect occurs in DRs within the 2 hour time frame. However, this result should be approached with caution as various forms (essential fatty acids, long chain fatty acids, and lysolipids) of arachidonic acid and docosahexaenoic acid were profiled in the discovery cohort and were not significantly different between ERs and DRs at post-challenge (Table S4). Other limiting factors of the validation cohort apart from the limited sample size include the use of various allergens (Table 2), and incomplete data on complete blood cell counts. The potency of different allergens is variable [54] and can dictate the type of response an individual may experience. For example, allergen inhalation challenge using house dust mite mainly elicits the dual response [55] (also seen in our data sets). Frequencies of white blood cell counts may influence plasma lysolipid levels and their metabolism can influence subsequent clinical phenotypes. In addition, measuring the specific arachidonic acid lysolipid (2-arachidonoylglycerophosphocholine) may be a more appropriate validation. However the differential expression of DHA may suggest that mechanisms involved in the resolution of inflammation may be significantly dysregulated between ERs and DRs resulting in their distinct clinical phenotypes.

To our knowledge, this study is the first of its kind to apply ‘omics’ methodologies to peripheral blood and identify molecular differences between two asthma phenotypes. These differences consist of factors associated with both systemic and airway inflammation. Although this study does not directly evaluate the impact of allergen inhalation on the airways, the downstream effects of this lung insult can provide significant evidence in understanding the molecular mechanisms underlying the early and late asthmatic responses. Our study has shown that careful selection of study participants is effective in detecting molecular differences between ERs and DRs. The use of multiple data sets from the same individuals can identify common biological functions and mechanisms and integration of these data sets may narrow down the number of generated hypotheses. Although many gene-metabolite clusters were identified, one biologically plausible gene-metabolite cluster was identified in DRs. Similar studies with larger sample sizes may reveal novel mechanisms, and targets for the pathogenesis and treatment of the late phase asthmatic response.

## Supporting Information

Figure S1 **Top biological functions and canonical pathways at pre-challenge.** The FDR, affy IDs and fold-change of genes were uploaded into IPA after which the following filters were applied: organ: lung, and cells: immune cells and FDR cut-off of 10%.
(DOCX)

Figure S2 **Top biological functions and canonical pathways for differentially expressed (FDR = 10%) genes at post-challenge.** (See Figure S1 for filtering criteria).
(DOCX)

Table S1 **Complete blood counts and differentials.** The Mean±SE of each cell count and differential at pre-challenge in early and dual responders. The Mean±SE of each cell count and differential at post-challenge (levels scaled to pre-challenge levels) in early and dual responders.
(DOCX)

Table S2 **Differentially expressed genes and metabolites at post-challenge (scaled to pre-challenge levels; post divided by pre levels).** A. Differentially expressed genes at post-challenge (FDR<10%). B. Differentially expressed metabolites at post-challenge (p-value<0.05).
(DOCX)

Table S3 **Differentially expressed lipids in the validation cohort.** Levels (Mean±SE) of arachidonic acid and docosahexaenoic at post-challenge (levels scaled to pre-challenge levels) in early and dual responders.
(DOCX)

Table S4 **Named metabolites profiled in this study.** Metabolites highlighted in red are differentially expressed at p<0.05.
(PDF)

## References

[1] Anderson GP (2008) Endotyping asthma: New insights into key pathogenic mechanisms in a complex, heterogeneous disease. Lancet 372: 1107–1119. 18805339 10.1016/S0140-6736(08)61452-X

[2] Tulic MK, Christodoulopoulos P, Hamid Q (2001) Small airway inflammation in asthma. Respir Res 2: 333–339. 11737932 10.1186/rr83 PMC64806

[3] Naylor B (1962) The shedding of the mucosa of the bronchial tree in asthma. Thorax 17: 69–72. 14478653 10.1136/thx.17.1.69 PMC1018672

[4] Tesfaigzi Y (2008) Regulation of mucous cell metaplasia in bronchial asthma. Curr Mol Med 8: 408–415. 18691068 10.2174/156652408785160961 PMC11503510

[5] Ebina M, Yaegashi H, Chiba R, Takahashi T, Motomiya M, et al (1990) Hyperreactive site in the airway tree of asthmatic patients revealed by thickening of bronchial muscles. A morphometric study. Am Rev Respir Dis 141: 1327–1332. 2187387 10.1164/ajrccm/141.5_Pt_1.1327

[6] Luskin AT (2005) What the asthma end points we know and love do and do not tell us. J Allergy Clin Immunol 115: S539–45. 15806037 10.1016/j.jaci.2005.01.027

[7] Gauvreau GM, Evans MY (2007) Allergen inhalation challenge: A human model of asthma exacerbation. Contrib Microbiol 14: 21–32. 17684330 10.1159/000107052

[8] Murray JJ, Tonnel AB, Brash AR, Roberts LJ,2nd, Gosset P, et al (1985) Prostaglandin D2 is released during acute allergic bronchospasm in man. Trans Assoc Am Physicians 98: 275–280. 3870298

[9] Liu MC, Hubbard WC, Proud D, Stealey BA, Galli SJ, et al (1991) Immediate and late inflammatory responses to ragweed antigen challenge of the peripheral airways in allergic asthmatics. cellular, mediator, and permeability changes. Am Rev Respir Dis 144: 51–58. 2064141 10.1164/ajrccm/144.1.51

[10] Gounni AS, Lamkhioued B, Ochiai K, Tanaka Y, Delaporte E, et al (1994) High-affinity IgE receptor on eosinophils is involved in defence against parasites. Nature 367: 183–186. 8114916 10.1038/367183a0

[11] Jarjour NN, Calhoun WJ, Kelly EA, Gleich GJ, Schwartz LB, et al (1997) The immediate and late allergic response to segmental bronchopulmonary provocation in asthma. Am J Respir Crit Care Med 155: 1515–1521. 9154851 10.1164/ajrccm.155.5.9154851

[12] Wenzel SE, Westcott JY, Smith HR, Larsen GL (1989) Spectrum of prostanoid release after bronchoalveolar allergen challenge in atopic asthmatics and in control groups. an alteration in the ratio of bronchoconstrictive to bronchoprotective mediators. Am Rev Respir Dis 139: 450–457. 2643903 10.1164/ajrccm/139.2.450

[13] Verstraelen S, Bloemen K, Nelissen I, Witters H, Schoeters G, et al (2008) Cell types involved in allergic asthma and their use in in vitro models to assess respiratory sensitization. Toxicol In Vitro 22: 1419–1431. 18603401 10.1016/j.tiv.2008.05.008

[14] Bousquet J, Jeffery PK, Busse WW, Johnson M, Vignola AM (2000) Asthma. From bronchoconstriction to airways inflammation and remodeling. Am J Respir Crit Care Med 161: 1720–1745. 10806180 10.1164/ajrccm.161.5.9903102

[15] Hansel NN, Diette GB (2007) Gene expression profiling in human asthma. Proc Am Thorac Soc 4: 32–36. 17202289 10.1513/pats.200606-132JG PMC2647611

[16] Adamko DJ, Sykes BD, Rowe BH (2012) The metabolomics of asthma: Novel diagnostic potential. Chest 141: 1295–1302. 22553262 10.1378/chest.11-2028

[17] Yao PL, Tsai MF, Lin YC, Wang CH, Liao WY, et al (2005) Global expression profiling of theophylline response genes in macrophages: Evidence of airway anti-inflammatory regulation. Respir Res 6: 89. 16083514 10.1186/1465-9921-6-89 PMC1215521

[18] Hakonarson H, Halapi E, Whelan R, Gulcher J, Stefansson K, et al (2001) Association between IL-1beta/TNF-alpha-induced glucocorticoid-sensitive changes in multiple gene expression and altered responsiveness in airway smooth muscle. Am J Respir Cell Mol Biol 25: 761–771. 11726403 10.1165/ajrcmb.25.6.4628

[19] Kam SH, Singh A, He JQ, Ruan J, Gauvreau GM, et al (2012) Peripheral blood gene expression changes during allergen inhalation challenge in atopic asthmatic individuals. J Asthma 49: 219–226. 22316092 10.3109/02770903.2011.654300

[20] Yamamoto M, Singh A, Ruan J, Gauvreau GM, O'Byrne PM, et al (2012) Decreased miR-192 expression in peripheral blood of asthmatic individuals undergoing an allergen inhalation challenge. BMC Genomics 13: 655–2164-13-655. 23170939 10.1186/1471-2164-13-655 PMC3598672

[21] Wood LJ, Inman MD, Watson RM, Foley R, Denburg JA, et al (1998) Changes in bone marrow inflammatory cell progenitors after inhaled allergen in asthmatic subjects. Am J Respir Crit Care Med 157: 99–105. 9445285 10.1164/ajrccm.157.1.9704125

[22] Gauvreau GM, Lee JM, Watson RM, Irani AM, Schwartz LB, et al (2000) Increased numbers of both airway basophils and mast cells in sputum after allergen inhalation challenge of atopic asthmatics. Am J Respir Crit Care Med 161: 1473–1478. 10806141 10.1164/ajrccm.161.5.9908090

[23] Matsumoto K, Gauvreau GM, Rerecich T, Watson RM, Wood LJ, et al (2002) IL-10 production in circulating T cells differs between allergen-induced isolated early and dual asthmatic responders. J Allergy Clin Immunol 109: 281–286. 11842298 10.1067/mai.2002.121144

[24] Singh A, Freue GV, Oosthuizen JL, Kam SH, Ruan J, et al.. (2012) Plasma proteomics can discriminate isolated early from dual responses in asthmatic individuals undergoing an allergen inhalation challenge. Proteomics Clin Appl. 10.1002/prca.201200013 22930592

[25] Hankinson JL, Odencrantz JR, Fedan KB (1999) Spirometric reference values from a sample of the general U.S. population. Am J Respir Crit Care Med 159: 179–187. 9872837 10.1164/ajrccm.159.1.9712108

[26] Cockcroft D, Murdock K, Mink J (1983) Determination of histamine PC20: Comparison of linear and logarithmic interpolation. Chest 84: 505–2. 6617292 10.1378/chest.84.4.505

[27] Gauvreau GM, Boulet LP, Cockcroft DW, Fitzgerald JM, Carlsten C, et al (2011) Effects of interleukin-13 blockade on allergen-induced airway responses in mild atopic asthma. Am J Respir Crit Care Med 183: 1007–1014. 21057005 10.1164/rccm.201008-1210OC

[28] Gautier L, Cope L, Bolstad BM, Irizarry RA (2004) Affy–analysis of affymetrix GeneChip data at the probe level. Bioinformatics 20: 307–315. 14960456 10.1093/bioinformatics/btg405

[29] Hochreiter S, Clevert DA, Obermayer K (2006) A new summarization method for affymetrix probe level data. Bioinformatics 22: 943–949. 16473874 10.1093/bioinformatics/btl033

[30] Talloen W, Clevert DA, Hochreiter S, Amaratunga D, Bijnens L, et al (2007) I/NI-calls for the exclusion of non-informative genes: A highly effective filtering tool for microarray data. Bioinformatics 23: 2897–2902. 17921172 10.1093/bioinformatics/btm478

[31] Bourgon R, Gentleman R, Huber W (2010) Independent filtering increases detection power for high-throughput experiments. PNAS 107: 9546–6. 20460310 10.1073/pnas.0914005107 PMC2906865

[32] Team RDC (2009) *R: A language and environment for statistical computing*.

[33] Folch J, Lees M, Sloane Stanley GH (1957) A simple method for the isolation and purification of total lipides from animal tissues. J Biol Chem 226: 497–509. 13428781

[34] Schlenk H, Gellerman JL (1960) Esterification of fatty acids with diazomethane on a small scale. Anal. Chem. 32: 1412–3.

[35] Symth GK (2004) Linear models and empirical bayes methods for assessing differential expression in microarrays experiments. Statistical Applications in Genetics and Molecular Biology Article 3. 10.2202/1544-6115.1027 16646809

[36] Benjamini Y, Hochberg Y (1995) Controlling the false discovery rate: A practical and powerful approach to multiple testing. 57: 289–12.

[37] Huber PJ (1981) Robust statistics. Wiley.

[38] Hotelling H (1936) Relations between two sets of variates. Biometrika 28: 321–57.

[39] Gonzalez I, Dejean S, Martin PGP, Goncalves O, Besse P, et al (2009) Highlight relationships between heterogeneous biological data through graphical displays based on regularized canonical correlation analysis. Journal of Biological Systems 17: 173–27.

[40] Lattka E, Illig T, Koletzko B, Heinrich J (2010) Genetic variants of the FADS1 FADS2 gene cluster as related to essential fatty acid metabolism. Curr Opin Lipidol 21: 64–69. 19809313 10.1097/MOL.0b013e3283327ca8

[41] Serhan CN, Chiang N, Van Dyke TE (2008) Resolving inflammation: Dual anti-inflammatory and pro-resolution lipid mediators. Nat Rev Immunol 8: 349–361. 18437155 10.1038/nri2294 PMC2744593

[42] Feltenmark S, Gautam N, Brunnstrom A, Griffiths W, Backman L, et al (2008) Eoxins are proinflammatory arachidonic acid metabolites produced via the 15-lipoxygenase-1 pathway in human eosinophils and mast cells. Proc Natl Acad Sci U S A 105: 680–685. 18184802 10.1073/pnas.0710127105 PMC2206596

[43] Levy BD (2010) Resolvins and protectins: Natural pharmacophores for resolution biology. Prostaglandins Leukot Essent Fatty Acids 82: 327–332. 20227865 10.1016/j.plefa.2010.02.003 PMC2896290

[44] Shindou H, Hishikawa D, Nakanishi H, Harayama T, Ishii S, et al (2007) A single enzyme catalyzes both platelet-activating factor production and membrane biogenesis of inflammatory cells. cloning and characterization of acetyl-CoA:LYSO-PAF acetyltransferase. J Biol Chem 282: 6532–6539. 17182612 10.1074/jbc.M609641200

[45] Wenzel SE (2012) Asthma phenotypes: The evolution from clinical to molecular approaches. Nat Med 18: 716–725. 22561835 10.1038/nm.2678

[46] Hamann J, Koning N, Pouwels W, Ulfman LH, van Eijk M, et al (2007) EMR1, the human homolog of F4/80, is an eosinophil-specific receptor. Eur J Immunol 37: 2797–2802. 17823986 10.1002/eji.200737553

[47] Seeds MC, Peachman KK, Bowton DL, Sivertson KL, Chilton FH (2009) Regulation of arachidonate remodeling enzymes impacts eosinophil survival during allergic asthma. Am J Respir Cell Mol Biol 41: 358–366. 19151322 10.1165/rcmb.2008-0192OC PMC2742755

[48] Rzehak P, Thijs C, Standl M, Mommers M, Glaser C, et al (2010) Variants of the FADS1 FADS2 gene cluster, blood levels of polyunsaturated fatty acids and eczema in children within the first 2 years of life. PLoS One 5: e13261. 20948998 10.1371/journal.pone.0013261 PMC2952585

[49] Chiang N, Arita M, Serhan CN (2005) Anti-inflammatory circuitry: Lipoxin, aspirin-triggered lipoxins and their receptor ALX. Prostaglandins Leukot Essent Fatty Acids 73: 163–177. 16125378 10.1016/j.plefa.2005.05.003

[50] Levy BD, Kohli P, Gotlinger K, Haworth O, Hong S, et al (2007) Protectin D1 is generated in asthma and dampens airway inflammation and hyperresponsiveness. J Immunol 178: 496–502. 17182589 10.4049/jimmunol.178.1.496 PMC3005704

[51] Perogamvros I, Ray DW, Trainer PJ (2012) Regulation of cortisol bioavailability–effects on hormone measurement and action. Nat Rev Endocrinol 8: 717–727. 22890008 10.1038/nrendo.2012.134

[52] Barnes PJ (1992) Bradykinin and asthma. Thorax 47: 979–983. 1465760 10.1136/thx.47.11.979 PMC464122

[53] Levy BD, Vachier I, Serhan CN (2012) Resolution of inflammation in asthma. Clin Chest Med 33: 559–570. 22929102 10.1016/j.ccm.2012.06.006 PMC3431599

[54] Hammad H, Lambrecht BN (2008) Dendritic cells and epithelial cells: Linking innate and adaptive immunity in asthma. Nat Rev Immunol 8: 193–204. 18301423 10.1038/nri2275

[55] Hatzivlassiou M, Grainge C, Kehagia V, Lau L, Howarth PH (2010) The allergen specificity of the late asthmatic reaction. Allergy 65: 355–358. 19804443 10.1111/j.1398-9995.2009.02184.x

