# Supplementary material for: Gene-Metabolite Expression in Blood Can Discriminate Allergen-Induced Isolated Early from Dual Asthmatic Responses
Source: PLoS One. 2013 Jul 2;8(7):e67907. doi: 10.1371/journal.pone.0067907 (PMC3699462; doi:10.1371/journal.pone.0067907)
Supplement: Figure S1 — Top biological functions and canonical pathways at pre-challenge. The FDR, affy IDs and fold-change of genes were uploaded into IPA after which the following filters were applied: organ: lung, and cells: immune cells and FDR cut-off of 10%. (DOCX) [file pone.0067907.s001.docx]

| **Biological Functions** | **Canonical Pathways** |
| --- | --- |
| 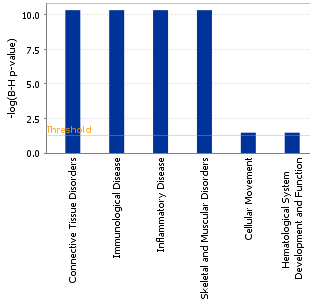 | 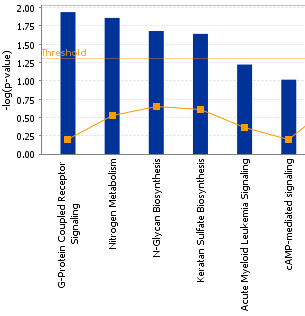 |

**Figure S1:** Top biological functions and canonical pathways at pre-challenge. The FDR, affy IDs and fold-change of genes were uploaded into IPA after which the following filters were applied: organ: lung, and cells: immune cells and FDR cut-off of 10%.
