# Supplementary material for: Gene-Metabolite Expression in Blood Can Discriminate Allergen-Induced Isolated Early from Dual Asthmatic Responses
Source: PLoS One. 2013 Jul 2;8(7):e67907. doi: 10.1371/journal.pone.0067907 (PMC3699462; doi:10.1371/journal.pone.0067907)
Supplement: Figure S2 — Top biological functions and canonical pathways for differentially expressed (FDR = 10%) genes at post-challenge. (See Figure S1 for filtering criteria). (DOCX) [file pone.0067907.s002.docx]

| **Biological Functions** | **Canonical Pathways** |
| --- | --- |
| 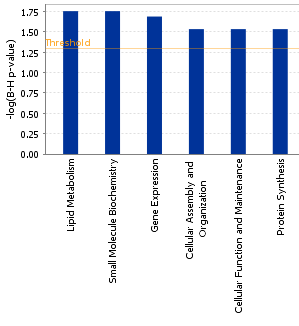 | 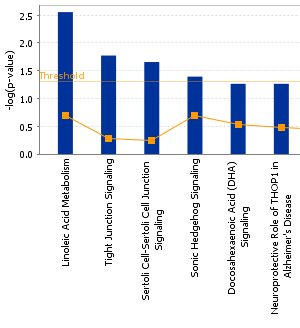 |

**Figure S2**: Top biological functions and canonical pathway s for differentially expressed (FDR=10%) genes at post-challenge (see Figure S1 for filtering criteria).
