# Supplementary material for: Gene-Metabolite Expression in Blood Can Discriminate Allergen-Induced Isolated Early from Dual Asthmatic Responses
Source: PLoS One. 2013 Jul 2;8(7):e67907. doi: 10.1371/journal.pone.0067907 (PMC3699462; doi:10.1371/journal.pone.0067907)
Supplement: Table S1 — Complete blood counts and differentials. The Mean±SE of each cell count and differential at pre-challenge in early and dual responders. The Mean±SE of each cell count and differential at post-challenge (levels scaled to pre-challenge levels) in early and dual responders. (DOCX) [file pone.0067907.s003.docx]

**Table S1: Complete blood counts and differentials**

|  | Pre-challenge | | | Post-challenge | | |
| --- | --- | --- | --- | --- | --- | --- |
|  | ER (Mean±SE) | DR (Mean±SE) | p-value | FC* in ER (Mean±SE) | FC in DR (Mean±SE) | p-value |
| Erythrocytes..(10^12^ cells/L) | 4.6±0.13 | 4.5±0.1 | 0.59 | -1.01±0.011 | -1.00±0.010 | 0.90 |
| Hemoglobin(g/L) | 137.5±3.43 | 133.8±4.94 | 0.31 | -1.01±0.009 | -1.00±0.009 | 0.83 |
| Hematocrit L/L | 0.4±0.01 | 0.4±0.02 | 0.57 | -1.01±0.011 | 1.00±0.010 | 0.97 |
| Mean.Cell.Volume (fL) | 87.5±1.73 | 88.3±1.87 | 0.77 | 1.00±0.004 | 1.00±0.004 | 0.84 |
| Mean.Cell.HB (pg/cell) | 29.9±0.68 | 29.8±0.65 | 0.76 | -1.00±0.004 | 1.00±0.005 | 0.89 |
| Mean.Cell.HB.Conc (g/L) | 342±4.87 | 337.3±1.96 | 0.57 | -1.01±0.003 | 1.00±0.005 | 0.41 |
| Red.Cell.Distr..Width (% CV) | 13.4±0.3 | 13.4±0.14 | 0.95 | -1.02±0.018 | 1.01±0.020 | 0.33 |
| Platelets (10^9^ cells/L) | 242±35.87 | 216±17.98 | 0.95 | 1.04±0.019 | 1.08±0.039 | 0.39 |
| Leukocytes (10^9^ cells/L) | 5.8±0.49 | 5±0.42 | 0.32 | 1.14±0.046 | 1.12±0.076 | 0.51 |
| Neutrophils (10^9^ cells/L) | 3.5±0.37 | 2.6±0.47 | 0.04 | 1.22±0.085 | 1.17±0.093 | 0.44 |
| Lymphocytes (10^9^ cells/L) | 1.6±0.2 | 1.9±0.2 | 0.13 | 1.07±0.059 | -1.02±0.059 | 0.40 |
| Monocytes (10^9^ cells/L) | 0.4±0.05 | 0.4±0.05 | 0.39 | -1.04±0.072 | -1.06±0.13 | 0.64 |
| Eosinophils (10^9^ cells/L) | 0.3±0.05 | 0.4±0.11 | 0.40 | -1.19±0.128 | -1.21±0.072 | 0.36 |
| Basophils (10^9^ cells/L) | 0.001±0 | 0±0.02 | 0.41 | ND† | ND | 0.15 |
| Relative.Neutrophils (%) | 0.6±0.03 | 0.5±0.04 | 0.01 | 1.07±0.035 | 1.12±0.066 | 0.69 |
| Relative.Lymphocytes (%) | 0.3±0.02 | 0.4±0.04 | 0.02 | -1.04±0.048 | -1.05±0.071 | 0.89 |
| Relative.Monocytes (%) | 0.1±0.01 | 0.1±0.01 | 0.84 | -1.15±0.058 | -1.28±0.099 | 0.37 |
| Relative.Eosinophils (%) | 0.05±0.01 | 0.1±0.01 | 0.07 | -1.38±0.076 | -1.15±0.073 | 0.72 |
| Relative.Basophils (%) | 0.004±0.001 | 0.01±0.003 | 0.12 | 1.12±0.152 | -1.27±0.200 | 0.31 |

*Levels at post-challenge are scaled to pre-challenge levels;

FC = post/pre if FC>0, FC = -1/(post/pre)

†ND (Not determined): division by zero cell-type frequency
