# Supplementary material for: Gene-Metabolite Expression in Blood Can Discriminate Allergen-Induced Isolated Early from Dual Asthmatic Responses
Source: PLoS One. 2013 Jul 2;8(7):e67907. doi: 10.1371/journal.pone.0067907 (PMC3699462; doi:10.1371/journal.pone.0067907)
Supplement: Table S2 — Differentially expressed genes and metabolites at post-challenge (scaled to pre-challenge levels; post divided by pre levels). A. Differentially expressed genes at post-challenge (FDR<10%). B. Differentially expressed metabolites at post-challenge (p-value<0.05). (DOCX) [file pone.0067907.s004.docx]

**Table S2. Differentially expressed genes and metabolites at post-challenge (scaled to pre-challenge levels, ratio of post to pre levels).**

**A. Differentially expressed genes in the interaction (FDR<0.1).**

|  |  | FC* in ERs | FC in DRs | P -Value | BH-FDR |
| --- | --- | --- | --- | --- | --- |
| 8011680 | ALOX15 | -1.034±0.053 | -1.007±0.013 | 1.37E-06 | 0.000681 |
| 8085062 | IL5RA | -1.072±0.008 | -1.022±0.005 | 5.80E-06 | 0.001 |
| 8124527 | HIST1H1B | -1.008±0.009 | 1.026±0.016 | 1.25E-05 | 0.002 |
| 7916862 | WLS | 1.011±0.011 | 1.036±0.018 | 1.38E-05 | 0.002 |
| 8047443 | STRADB | -1.021±0.007 | 1.011±0.005 | 0.000193 | 0.019 |
| 7940565 | FADS2 | -1.032±0.013 | 1.015±0.008 | 0.000254 | 0.020 |
| 7995697 | LPCAT2 | -1.009±0.005 | 1.015±0.011 | 0.00028 | 0.020 |
| 8161288 | CNTNAP3 | 1.017±0.012 | 1.058±0.024 | 0.000422 | 0.026 |
| 7953901 | CLEC12A | -1.030±0.015 | 1.018±0.018 | 0.000609 | 0.034 |
| 8166632 | GK | 1.007±0.011 | 1.029±0.018 | 0.00087 | 0.043 |
| 8174361 | TSC22D3 | -1.037±0.006 | -1.014±0.011 | 0.001 | 0.046 |
| 8169365 | TMEM164 | -1.004±0.006 | 1.016±0.007 | 0.001 | 0.046 |
| 8176709 | CYorf15B | -1.003±0.018 | 1.028±0.038 | 0.001 | 0.050 |
| 8007607 | RUNDC3A | -1.021±0.005 | 1.014±0.019 | 0.001 | 0.050 |
| 8161460 | CNTNAP3 | 1.037±0.015 | 1.081±0.032 | 0.002 | 0.058 |
| 8025103 | EMR1 | -1.040±0.005 | -1.007±0.009 | 0.002 | 0.063 |
| 7988033 | EPB42 | -1.028±0.008 | 1.015±0.010 | 0.002 | 0.063 |
| 8164596 | C9orf78 | -1.016±0.007 | 1.003±0.002 | 0.002 | 0.063 |
| 7921873 | FCGR3A | 1.011±0.007 | 1.020±0.011 | 0.003 | 0.084 |
| 8153652 | SHARPIN | -1.024±0.008 | 1.009±0.007 | 0.004 | 0.087 |
| 8135378 | PRKAR2B | -1.015±0.008 | 1.021±0.005 | 0.004 | 0.087 |
| 7961230 | CSDA | -1.027±0.007 | 1.008±0.009 | 0.004 | 0.093 |
| 7929052 | IFIT3 | 1.000±0.01 | 1.033±0.023 | 0.004 | 0.093 |
| 8171834 | RPL9 | 1.022±0.02 | -1.029±0.010 | 0.005 | 0.093 |
| 8157264 | SLC31A2 | -1.019±0.005 | 1.009±0.012 | 0.005 | 0.093 |

*Levels at post-challenge are scaled to pre-challenge levels;

FC = post/pre if FC>0, FC = -1/(post/pre)

B. **Differentially expressed metabolites at post-challenge (p-value<0.05).**

| BIOCHEMICAL | SUB_PATHWAY | HMDB | FC* in ERs* | FC in DRs | P -Value |
| --- | --- | --- | --- | --- | --- |
| andro steroid monosulfate 1* | Sterol/Steroid | HMDB02759 | 1.003±0.120 | 1.054±0.202 | 0.002 |
| 4-hydroxyphenylacetate | Phenylalanine & tyrosine metabolism | HMDB00020 | -1.519±0.210 | 1.226±0.414 | 0.006 |
| thymol sulfate | Food component/Plant | HMDB01878 | -1.085±0.146 | -1.381±0.317 | 0.014 |
| 1-pentadecanoylglycerophosphocholine* | Lysolipid |  | 1.404±0.569 | -1.005±0.300 | 0.014 |
| 2-arachidonoylglycerophosphocholine* | Lysolipid |  | 3.950±2.892 | 1.181±0.436 | 0.014 |
| cortisol | Sterol/Steroid | HMDB00063 | -1.560±0.210 | 1.008±0.400 | 0.017 |
| 3-methyl-2-oxobutyrate | Valine, leucine and isoleucine metabolism | HMDB00019 | -1.048±0.407 | 1.307±0.341 | 0.018 |
| 1-linoleoylglycerophosphocholine | Lysolipid |  | 1.046±0.192 | 1.185±0.255 | 0.019 |
| 7-alpha-hydroxy-3-oxo-4-cholestenoate (7-Hoca) | Sterol/Steroid | HMDB12458 | 1.188±0.207 | 1.416±0.253 | 0.031 |
| cysteine | Cysteine, methionine, SAM, taurine metabolism | HMDB00574 | -1.037±0.215 | -1.404±0.110 | 0.041 |
| bradykinin, hydroxy-pro(3) | Polypeptide | HMDB11728 | -1.344±0.241 | 2.788±3.447 | 0.043 |

*Levels at post-challenge are scaled to pre-challenge levels;

FC = post/pre if FC>0, FC = -1/(post/pre)
