# Supplementary material for: Gene-Metabolite Expression in Blood Can Discriminate Allergen-Induced Isolated Early from Dual Asthmatic Responses
Source: PLoS One. 2013 Jul 2;8(7):e67907. doi: 10.1371/journal.pone.0067907 (PMC3699462; doi:10.1371/journal.pone.0067907)
Supplement: Table S3 — Differentially expressed lipids in the validation cohort. Levels (Mean±SE) of arachidonic acid and docosahexaenoic at post-challenge (levels scaled to pre-challenge levels) in early and dual responders. (DOCX) [file pone.0067907.s005.docx]

**Table S3: Differentially expressed lipids in the validation cohort.**

|  | FC* in ERs | FC in DRs | P -Value |
| --- | --- | --- | --- |
| DHA free (nmoles./µgram.of.protein) | -1.10±0.04 | 1.02±0.04 | 0.03 |
| AA phospholipid (nmoles./µgram.of.protein) | -1.08±0.05 | 1.02±0.06 | 0.21 |
| AA free (nmoles./µgram.of.protein) | 1.22±0.20 | 1.41±0.12 | 0.59 |
| DHA phospholipid (nmoles./µgram.of.protein) | 1.05±0.06 | 1.02±0.06 | 0.63 |

*Levels at post-challenge are scaled to pre-challenge levels;

FC = post/pre if FC>0, FC = -1/(post/pre)
