# Supplementary material for: Gene-Metabolite Expression in Blood Can Discriminate Allergen-Induced Isolated Early from Dual Asthmatic Responses
Source: PLoS One. 2013 Jul 2;8(7):e67907. doi: 10.1371/journal.pone.0067907 (PMC3699462; doi:10.1371/journal.pone.0067907)
Supplement: Table S4 — Named metabolites profiled in this study. Metabolites highlighted in red are differentially expressed at p<0.05. (PDF) [file pone.0067907.s006.pdf]

Named metabolites profiled in this study. **Shaded cells indicate $p \leq 0.05$**

PATHWAY SORT	SUPER PATHWAY	SUB PATHWAY	BIOCHEMICAL NAME	PLATFORM	COMP ID	KEGG	HMDB	Pre-challenge	Post-challenge
1	Amino acid	Glycine, serine and threonine metabolism	glycine	GC/MS	32338	C00037	HMDB00123	0.9773	0.1069
3			dimethylglycine	GC/MS	5086	C01026	HMDB00092	0.8356	0.0759
4			N-acetylglycine	GC/MS	27710		HMDB00532	0.0224	0.0621
5			N-formylglycine	GC/MS	1596			0.3479	0.1218
6			beta-hydroxypyruvate	GC/MS	15686	C00168	HMDB01352	0.6966	0.5392
7			serine	GC/MS	32315	C00065	HMDB03406	0.3766	0.8752
9			N-acetylserine	GC/MS	37076		HMDB02931	0.9043	0.3175
16			threonine	LC/MS pos	1284	C00188	HMDB00167	0.2985	0.6786
20			N-(2-furoyl)glycine	LC/MS pos	31536		HMDB00439	0.1544	0.1544
21			betaine	LC/MS pos	3141	C00719	HMDB00043	0.4175	0.9807
23		Alanine and aspartate metabolism	alanine	GC/MS	32339	C00041	HMDB00161	0.9031	0.7872
24			beta-alanine	GC/MS	35838	C00099	HMDB00056	0.2604	0.0579
26			N-acetylalanine	LC/MS neg	1585	C02847	HMDB00766	0.2504	0.4600
27			N-acetyl-beta-alanine	LC/MS pos	37432	C01073		0.2991	0.4536
29			aspartate	GC/MS	35834	C00049	HMDB00191	0.7561	0.8588
33			asparagine	GC/MS	34283	C00152	HMDB00168	0.5249	0.9715
38		Glutamate metabolism	glutamate	GC/MS	32322	C00025	HMDB03339	0.5697	0.5616
42			glutamine	LC/MS pos	53	C00064	HMDB00641	0.3835	0.8355
43			pyroglutamine*	LC/MS pos	32672			0.0830	0.7976
48		Histidine metabolism	histidine	LC/MS neg	59	C00135	HMDB00177	0.8068	0.4715
51			3-methylhistidine	LC/MS neg	15677	C01152	HMDB00479	0.8158	0.7155
66		Lysine metabolism	lysine	GC/MS	35836	C00047	HMDB00182	0.6664	0.4579
69			pipecolate	LC/MS pos	1444	C00408	HMDB00070	0.5233	0.4734
74			N6-acetyllysine	LC/MS pos	36752	C02727	HMDB00206	0.6733	0.8080
75			glutaroyl carnitine	LC/MS pos	35439		HMDB13130	0.9684	0.0910
77		Phenylalanine & tyrosine metabolism	phenyllactate (PLA)	LC/MS neg	22130	C05607	HMDB00779	0.2980	0.4201
78			phenylalanine	LC/MS pos	64	C00079	HMDB00159	0.2444	0.9616
80			phenylacetate	LC/MS neg	15958	C07086	HMDB00209	0.4940	0.5339
82			p-cresol sulfate	LC/MS neg	36103	C01468		0.9233	0.5269
90			tyrosine	LC/MS pos	1299	C00082	HMDB00158	0.2655	0.3479
91			3-(4-hydroxyphenyl)lactate	LC/MS neg	32197	C03672	HMDB00755	0.8469	0.1321
97			4-hydroxyphenylpyruvate	LC/MS neg	1669	C01179	HMDB00707	0.8648	0.6273
106			4-hydroxyphenylacetate	GC/MS	541	C00642	HMDB00020	0.9462	0.0055
110			3-methoxytyrosine	LC/MS pos	12017		HMDB01434	0.8608	0.9903
115			phenylacetylglutamine	LC/MS pos	35126	C05597	HMDB06344	0.8936	0.4842
119			3-phenylpropionate (hydrocinnamate)	LC/MS neg	15749	C05629	HMDB00764	0.8407	0.9535
121			phenol sulfate	LC/MS neg	32553	C02180		0.6961	0.0977
125		Tryptophan metabolism	kynurenine	LC/MS pos	15140	C00328	HMDB00684	0.6616	0.8830
126			tryptophan	LC/MS pos	54	C00078	HMDB00929	0.3733	0.7068
127			indolelactate	GC/MS	18349	C02043	HMDB00671	0.0827	0.2156
129			indoleacetate	LC/MS pos	27513	C00954	HMDB00197	0.1009	0.7881
131			tryptophan betaine	LC/MS pos	37097	C09213		0.7485	0.5376
138			serotonin (5HT)	LC/MS pos	2342	C00780	HMDB00259	0.1883	0.3511
141			C-glycosyltryptophan*	LC/MS pos	32675			0.8916	0.3842
147			3-indoxyl sulfate	LC/MS neg	27672		HMDB00682	0.4038	0.6606
148			indolepropionate	GC/MS	32405		HMDB02302	0.9499	0.5636
149			3-methyl-2-oxobutyrate	LC/MS neg	21047	C00141	HMDB00019	0.0554	0.0180
150			3-methyl-2-oxovalerate	LC/MS neg	15676	C00671	HMDB03736	0.3628	0.6419
155			beta-hydroxyisovalerate	LC/MS neg	12129		HMDB00754	0.4131	0.9643
156			alpha-hydroxyisocaproate	GC/MS	22132	C03264	HMDB00746	0.4949	0.1493
157			isoleucine	LC/MS pos	1125	C00407	HMDB00172	0.3452	0.3935

158		Valine, leucine and isoleucine metabolism	leucine	LC/MS pos	60	C00123	HMDB00687	0.0520	0.1468
167			valine	LC/MS pos	1649	C00183	HMDB00883	0.1200	0.1324
168			2-hydroxyisobutyrate	GC/MS	22030		HMDB00729	0.7945	0.5942
170			4-methyl-2-oxopentanoate	LC/MS neg	22116	C00233	HMDB00695	0.2985	0.2668
175			alpha-hydroxyisovalerate	GC/MS	33937		HMDB00407	0.9553	0.6551
177			isobutyrylcarnitine	LC/MS pos	33441		HMDB00736	0.2272	0.9941
179			2-methylbutyroylcarnitine	LC/MS pos	35431		HMDB00378	0.6827	0.6512
181			isovalerylcarnitine	LC/MS pos	34407		HMDB00688	0.1703	0.8730
182			hydroxyisovaleroyl carnitine	LC/MS pos	35433			0.9060	0.8655
188		Cysteine, methionine, SAM, taurine metabolism	cysteine	GC/MS	31453	C00097	HMDB00574	0.1033	0.0410
194			N-formylmethionine	LC/MS neg	2829	C03145	HMDB01015	0.9585	0.8707
199			methionine	LC/MS pos	1302	C00073	HMDB00696	0.0039	0.1310
201			N-acetylmethionine	LC/MS neg	1589	C02712	HMDB11745	0.7183	0.9075
202			alpha-ketobutyrate	LC/MS neg	4968	C00109	HMDB00005	0.5096	0.8342
203			2-hydroxybutyrate (AHB)	GC/MS	21044	C05984	HMDB00008	0.2699	0.7103
213		Urea cycle; arginine-, proline-, metabolism	arginine	LC/MS neg	1638	C00062	HMDB00517	0.6091	0.9850
216			ornithine	GC/MS	35832	C00077	HMDB03374	0.5529	0.8294
217			urea	GC/MS	1670	C00086	HMDB00294	0.6564	0.8730
218			proline	LC/MS pos	1898	C00148	HMDB00162	0.6887	0.9377
220			citrulline	LC/MS pos	2132	C00327	HMDB00904	0.8528	0.1334
221			N-acetylornithine	LC/MS pos	15630	C00437	HMDB03357	0.3731	0.5547
224			trans-4-hydroxyproline	GC/MS	32319	C01157	HMDB00725	0.5184	0.7295
232		Creatine metabolism	creatine	LC/MS pos	27718	C00300	HMDB00064	0.9177	0.5397
233			creatinine	LC/MS pos	513	C00791	HMDB00562	0.3472	0.1890
235		Butanoate metabolism	2-aminobutyrate	GC/MS	32309	C02261	HMDB00650	0.8138	0.8277
247		Guanidino and acetamido metabolism	4-acetamidobutanoate	LC/MS pos	1558	C02946	HMDB03681	0.4512	0.9619
253		Glutathione metabolism	5-oxoproline	LC/MS neg	1494	C01879	HMDB00267	0.2157	0.2836
298	Peptide	Dipeptide	pro-hydroxy-pro	LC/MS pos	35127		HMDB06695	0.7856	0.2311
321		gamma-glutamyl	gamma-glutamylvaline	LC/MS pos	32393		HMDB11172	0.5871	0.8673
323			gamma-glutamylleucine	LC/MS pos	18369		HMDB11171	0.8830	0.3906
324			gamma-glutamylisoleucine*	LC/MS pos	34456		HMDB11170	0.3643	0.6019
327			gamma-glutamylmethionine	LC/MS pos	37539			0.1446	0.6047
331			gamma-glutamylphenylalanine	LC/MS pos	33422		HMDB00594	0.3695	0.2189
332			gamma-glutamyltyrosine	LC/MS pos	2734			0.2090	0.8757
336			gamma-glutamylalanine	LC/MS pos	37063			0.3655	0.6283
337		Polypeptide	bradykinin	LC/MS pos	22154	C00306	HMDB04246	0.3800	0.0547
338			bradykinin, hydroxy-pro(3)	LC/MS pos	33962		HMDB11728	0.7089	0.0429
339			bradykinin, des-arg(9)	LC/MS pos	34420	C00306	HMDB04246	0.1848	0.3271
343			HWESASXX*	LC/MS pos	32836			0.4784	0.2194
345			[H]HWESASLLR[OH]	LC/MS pos	33964			0.8241	0.4578
354		Fibrinogen cleavage peptide	ADSGEGDFXAEGGGVR*	LC/MS pos	33084			0.3214	0.2859
372	Carbohydrate	Aminosugars metabolism	erythronate*	GC/MS	33477		HMDB00613	0.4497	0.1625
389		Fructose, mannose, galactose, starch, and sucrose metabolism	fructose	GC/MS	31266	C00095	HMDB00660	0.9890	0.3352
398			mannitol	GC/MS	15335	C00392	HMDB00765	0.8050	0.9718
402			mannose	GC/MS	584	C00159	HMDB00169	0.0403	0.1649
430		Glycolysis, gluconeogenesis, pyruvate metabolism	1,5-anhydroglucitol (1,5-AG)	GC/MS	20675	C07326	HMDB02712	0.5548	0.4131
432			glycerate	GC/MS	1572	C00258	HMDB00139	0.1548	0.4634
436			glucose	GC/MS	20488	C00293	HMDB00122	0.3029	0.4384
444			3-phosphoglycerate	GC/MS	1414	C00597	HMDB00807	0.3059	0.6315
450			pyruvate	GC/MS	599	C00022	HMDB00243	0.4391	0.2392
451			lactate	GC/MS	527	C00186	HMDB00190	0.3466	0.9505
463		Nucleotide sugars, pentose metabolism	threitol	GC/MS	35854	C16884	HMDB04136	0.1165	0.6685
480			arabinose	GC/MS	575	C00181	HMDB00646	0.0599	0.3859

483		metabolism	xylose	GC/MS	15835	C00181	HMDB00098	0.1063	0.8267
491			citrate	GC/MS	1564	C00158	HMDB00094	0.6547	0.6418
500			alpha-ketoglutarate	GC/MS	33453	C00026	HMDB00208	0.5142	0.5710
501			succinate	GC/MS	1437	C00042	HMDB00254	0.1856	0.9019
502		Krebs cycle	succinylcarnitine	LC/MS pos	37058			0.1438	0.9271
504			fumarate	GC/MS	1643	C00122	HMDB00134	0.7652	0.4707
507	Energy		malate	GC/MS	1303	C00149	HMDB00156	0.0391	0.3967
508			oxaloacetate	GC/MS	35252	C00036	HMDB00223	0.6949	0.3560
510			acetylphosphate	GC/MS	15488	C00227	HMDB01494	0.0927	0.1210
511		Oxidative phosphorylation	phosphate	GC/MS	11438	C00009	HMDB01429	0.3211	0.1994
512			pyrophosphate (PPi)	GC/MS	2078	C00013	HMDB00250	0.1891	0.5469
512.5			linoleate (18:2n6)	LC/MS neg	1105	C01595	HMDB00673	0.7682	0.7085
516			linolenate [alpha or gamma; (18:3n3 or 6)]	LC/MS neg	34035	C06427	HMDB01388	0.9461	0.5141
517			dihomo-linolenate (20:3n3 or n6)	LC/MS neg	35718	C03242	HMDB02925	0.7007	0.6917
518		Essential fatty acid	eicosapentaenoate (EPA; 20:5n3)	LC/MS neg	18467	C06428	HMDB01999	0.7624	0.4712
519			docosapentaenoate (n3 DPA; 22:5n3)	LC/MS neg	32504	C16513	HMDB01976	0.3751	0.8930
520			docosapentaenoate (n6 DPA; 22:5n6)	LC/MS neg	37478	C06429	HMDB13123	0.7621	0.9600
521			docosahexaenoate (DHA; 22:6n3)	LC/MS neg	19323	C06429	HMDB02183	0.3600	0.9474
525			caproate (6:0)	LC/MS neg	32489	C01585	HMDB00535	0.9521	0.3943
526			heptanoate (7:0)	LC/MS neg	1644	C17714	HMDB00666	0.7261	0.4266
527			caprylate (8:0)	LC/MS neg	32492	C06423	HMDB00482	0.1309	0.2609
528		Medium chain fatty acid	pelargonate (9:0)	LC/MS neg	12035	C01601	HMDB00847	0.1613	0.9166
529			caprate (10:0)	LC/MS neg	1642	C01571	HMDB00511	0.0846	0.3541
530			undecanoate (11:0)	LC/MS neg	12067		HMDB00947	0.5550	0.7635
532			laurate (12:0)	LC/MS neg	1645	C02679	HMDB00638	0.5890	0.9480
533			5-dodecenoate (12:1n7)	LC/MS neg	33968		HMDB00529	0.8778	0.8268
535			myristate (14:0)	LC/MS neg	1365	C06424	HMDB00806	0.8935	0.8214
536			myristoleate (14:1n5)	LC/MS neg	32418	C08322	HMDB02000	0.9724	0.8216
537			pentadecanoate (15:0)	GC/MS	1361	C16537	HMDB00826	0.3042	0.3084
538			palmitate (16:0)	LC/MS neg	1336	C00249	HMDB00220	0.6613	0.6888
539			palmitoleate (16:1n7)	LC/MS neg	33447	C08362	HMDB03229	0.8542	0.6879
541			margarate (17:0)	LC/MS neg	1121		HMDB02259	0.6536	0.8285
542			10-heptadecenoate (17:1n7)	LC/MS neg	33971			0.8764	0.8806
543		Long chain fatty acid	stearate (18:0)	LC/MS neg	1358	C01530	HMDB00827	0.7956	0.8359
545			oleate (18:1n9)	LC/MS neg	1359	C00712	HMDB00207	0.6821	0.6317
552			stearidonate (18:4n3)	LC/MS neg	33969	C16300	HMDB06547	0.8244	0.7118
554			10-nonadecenoate (19:1n9)	LC/MS neg	33972			0.8642	0.8754
559			eicosenoate (20:1n9 or 11)	LC/MS neg	33587		HMDB02231	0.8121	0.4688
561			dihomo-linoleate (20:2n6)	LC/MS neg	17805	C16525		0.5942	0.8796
565			arachidonate (20:4n6)	LC/MS neg	1110	C00219	HMDB01043	0.3492	0.5287
569			docosadienoate (22:2n6)	LC/MS neg	32415	C16533		0.4470	0.4746
571			adrenate (22:4n6)	LC/MS neg	32980	C16527	HMDB02226	0.2385	0.9591
594			3-hydroxyoctanoate	LC/MS neg	22001		HMDB01954	0.5509	0.5146
602		Fatty acid, monohydroxy	2-hydroxystearate	LC/MS neg	17945	C03045		0.2663	0.9601
604			2-hydroxypalmitate	LC/MS neg	35675			0.6356	0.6455
610			2-hydroxyglutarate	GC/MS	37253	C02630	HMDB00606	0.2886	0.8328
616			azelate (nonanedioate)	LC/MS neg	18362	C08261	HMDB00784	0.5695	0.3440
617			dodecanedioate	LC/MS neg	32388	C02678	HMDB00623	0.9032	0.2109
618		Fatty acid, dicarboxylate	tetradecanedioate	LC/MS neg	35669		HMDB00872	0.2839	0.8029
619			hexadecanedioate	LC/MS neg	35678		HMDB00672	0.0568	0.6005
620			octadecanedioate	LC/MS neg	36754		HMDB00782	0.1449	0.4704
622			3-carboxy-4-methyl-5-propyl-2-furanpropanoate (CMPF)	LC/MS neg	31787			0.4614	0.3934
632.65		Fatty acid, branched	methyl palmitate (15 or 2)	LC/MS neg	38768			0.0045	0.3873
632.7			17-methylstearate	LC/MS neg	38296			0.7361	0.9754
683		Fatty acid metabolism (also	propionylcarnitine	LC/MS pos	32452	C03017	HMDB00824	0.5445	0.9620

685		BCAA metabolism)	butyrylcarnitine	LC/MS pos	32412	C02862	HMDB02013	0.4105	0.3262
689		Fatty acid metabolism	isovalerate	LC/MS neg	34732	C08262	HMDB00718	0.3983	0.9205
691			deoxycarnitine	LC/MS pos	36747	C01181	HMDB01161	0.9535	0.2169
692			carnitine	LC/MS pos	15500	C00487	HMDB00062	0.6641	0.2499
693			3-dehydrocarnitine*	LC/MS pos	32654	C02636	HMDB12154	0.9160	0.7743
694			acetylcarnitine	LC/MS pos	32198	C02571	HMDB00201	0.9495	0.4166
696			hexanoylcarnitine	LC/MS pos	32328	C01585	HMDB00705	0.3508	0.8426
698		Carnitine metabolism	octanoylcarnitine	LC/MS pos	33936	C02838	HMDB00791	0.2684	0.6043
700			decanoylcarnitine	LC/MS pos	33941	C03299	HMDB00651	0.3632	0.3629
701.5			cis-4-decenoyl carnitine	LC/MS pos	38178			0.5578	0.4604
702			laurylcarnitine	LC/MS pos	34534		HMDB02250	0.1741	0.2557
705			palmitoylcarnitine	LC/MS pos	22189	C02990	HMDB00222	0.5745	0.2505
707			oleoylcarnitine	LC/MS pos	35160		HMDB05065	0.5822	0.8518
709			cholate	LC/MS neg	22842	C00695	HMDB00619	0.3594	0.9987
712	Lipid		glycocholate	LC/MS neg	18476	C01921	HMDB00138	0.7774	0.5188
719			deoxycholate	LC/MS neg	1114	C04483	HMDB00626	0.2636	0.8695
723		Bile acid metabolism	glycochenodeoxycholate	LC/MS neg	32346	C05466	HMDB00637	0.7799	0.6578
725			glycolithocholate sulfate*	LC/MS neg	32620	C11301	HMDB02639	0.1897	0.7785
727			taurolithocholate 3-sulfate	LC/MS neg	36850	C03642	HMDB02580	0.9284	0.4337
741			glycocholenate sulfate*	LC/MS neg	32599			0.8451	0.5513
742			taurocholenate sulfate*	LC/MS neg	32807			0.2133	0.3040
742.1			glycoursodeoxycholate	LC/MS neg	39379		HMDB00708	0.9403	0.6737
750			glycerol	GC/MS	15122	C00116	HMDB00131	0.5552	0.7552
751		Glycerolipid metabolism	choline	LC/MS pos	15506	C00114	HMDB00097	0.6618	0.3496
752			glycerol 3-phosphate (G3P)	GC/MS	15365	C00093	HMDB00126	0.1960	0.6379
753			glycerophosphorylcholine (GPC)	LC/MS pos	15990	C00670	HMDB00086	0.2295	0.7966
760		Inositol metabolism	myo-inositol	GC/MS	19934	C00137	HMDB00211	0.9573	0.8610
761			chiro-inositol	GC/MS	37112			0.2569	0.8320
769			3-hydroxybutyrate (BHBA)	GC/MS	542	C01089	HMDB00357	0.2980	0.5416
771		Ketone bodies	1,2-propanediol	GC/MS	38002	C00717,C02912,C00583,C01506,C02917	HMDB01881	0.9966	0.8412
775			1-palmitoylglycerophosphoethanolamine	LC/MS neg	35631		HMDB11503	0.3341	0.4953
776			2-palmitoylglycerophosphoethanolamine*	LC/MS neg	35688			0.4160	0.6591
780			1-stearoylglycerophosphoethanolamine	LC/MS neg	34416		HMDB11130	0.2123	0.9103
781			1-oleoylglycerophosphoethanolamine	LC/MS neg	35628		HMDB11506	0.1014	0.8832
782			2-oleoylglycerophosphoethanolamine*	LC/MS neg	35687			0.5255	0.3438
783			1-linoleoylglycerophosphoethanolamine*	LC/MS neg	32635		HMDB11507	0.1848	0.7754
784			2-linoleoylglycerophosphoethanolamine*	LC/MS neg	36593			0.2086	0.5761
785			1-arachidonoylglycerophosphoethanolamine*	LC/MS neg	35186		HMDB11517	0.4125	0.9407
786			2-arachidonoylglycerophosphoethanolamine*	LC/MS neg	32815			0.7576	0.3346
791			1-myristoylglycerophosphocholine	LC/MS pos	35626		HMDB10379	0.8213	0.2310
793			1-pentadecanoylglycerophosphocholine*	LC/MS pos	37418			0.3997	0.0142
794			1-palmitoylglycerophosphocholine	LC/MS pos	33955			0.1798	0.1368
795			2-palmitoylglycerophosphocholine*	LC/MS pos	35253			0.6198	0.3514
796		Lysolipid	1-palmitoleoylglycerophosphocholine*	LC/MS pos	33230			0.9632	0.6232
798			1-heptadecanoylglycerophosphocholine	LC/MS pos	33957		HMDB12108	0.7068	0.4604
799			1-stearoylglycerophosphocholine	LC/MS pos	33961			0.3368	0.1491
800			2-stearoylglycerophosphocholine*	LC/MS pos	35255			0.4747	0.6119
801			1-oleoylglycerophosphocholine	LC/MS pos	33960			0.5362	0.1938
802			2-oleoylglycerophosphocholine*	LC/MS pos	35254			0.9242	0.8482
803			1-linoleoylglycerophosphocholine	LC/MS pos	34419	C04100		0.0834	0.0194
804			2-linoleoylglycerophosphocholine*	LC/MS neg	38087			0.1441	0.8527
806			1-eicosadienoylglycerophosphocholine*	LC/MS pos	33871			0.6331	0.4013
807			1-eicosatrienoylglycerophosphocholine*	LC/MS pos	33821			0.3641	0.5863
809			1-arachidonoylglycerophosphocholine*	LC/MS pos	33228	C05208		0.7989	0.3767
810			2-arachidonoylglycerophosphocholine*	LC/MS pos	35256			0.0035	0.0143
811			1-docosapentaenoylglycerophosphocholine*	LC/MS pos	37231			0.6429	0.3310
813			1-docosahexaenoylglycerophosphocholine*	LC/MS pos	33822			0.8332	0.7376
818			1-stearoylglycerophosphoinositol	LC/MS neg	19324			0.3272	0.6597
821			1-arachidonoylglycerophosphoinositol*	LC/MS neg	34214			0.2258	0.3443
825.5			1-palmitoylplasmenylethanolamine*	LC/MS neg	39270			0.2408	0.1363

864		Sphingolipid	palmitoyl sphingomyelin	GC/MS	37506			0.6271	0.6529
865			stearoyl sphingomyelin	GC/MS	19503	C00550	HMDB01348	0.6340	0.5647
881		Sterol/Steroid	cholesterol	GC/MS	63	C00187	HMDB00067	0.1719	0.7538
891			dehydroisoandrosterone sulfate (DHEA-S)	LC/MS neg	32425	C04555	HMDB01032	0.2637	0.7153
892			epiandrosterone sulfate	LC/MS neg	33973	C07635	HMDB00365	0.9708	0.5426
895			androsterone sulfate	LC/MS neg	31591	C00523	HMDB02759	0.4674	0.9342
912			cortisol	LC/MS pos	1712	C00735	HMDB00063	0.1519	0.0172
914			cortisone	LC/MS pos	1769	C00762	HMDB02802	0.3326	0.7336
934			7-alpha-hydroxy-3-oxo-4-cholestenoate (7-Hoca)	LC/MS neg	36776	C17337	HMDB12458	0.1507	0.0307
935			4-androsten-3beta,17beta-diol disulfate 1*	LC/MS neg	37202		HMDB03818	0.4169	0.5788
936			4-androsten-3beta,17beta-diol disulfate 2*	LC/MS neg	37203		HMDB03818	0.6962	0.1373
937			5alpha-androstan-3beta,17beta-diol disulfate	LC/MS neg	37190		HMDB00493	0.4365	0.7424
938			5alpha-pregnan-3beta,20alpha-diol disulfate	LC/MS neg	37198			0.6946	0.2313
940			pregnen-diol disulfate*	LC/MS neg	32562	C05484	HMDB04025	0.4167	0.1645
941			pregn steroid monosulfate*	LC/MS neg	32619	C18044	HMDB00774	0.8218	0.1556
942			andro steroid monosulfate 1*	LC/MS neg	32827	C04555	HMDB02759	0.4867	0.0019
943			andro steroid monosulfate 2*	LC/MS neg	32792	C04555	HMDB02759	0.8246	0.8564
944			21-hydroxypregnenolone disulfate	LC/MS neg	37173	C05485	HMDB04026	0.4763	0.1523
946.4			pregnenolone sulfate	LC/MS neg	38170		HMDB00774	0.7701	0.4721
950	Nucleotide	Purine metabolism, (hypo)xanthine/inosine containing	hypoxanthine	GC/MS	3127	C00262	HMDB00157	0.9266	0.3391
958		Purine metabolism, adenine containing	N1-methyladenosine	LC/MS pos	15650	C02494	HMDB03331	0.6718	0.9425
994		Purine metabolism, urate metabolism	urate	LC/MS neg	1604	C00366	HMDB00289	0.5912	0.6914
995			allantoin	GC/MS	1107	C02350	HMDB00462	0.5811	0.7090
1024		Pyrimidine metabolism, uracil containing	uridine	LC/MS neg	606	C00299	HMDB00296	0.4419	0.9065
1025			pseudouridine	LC/MS neg	33442	C02067	HMDB00767	0.8546	0.0953
1036		Purine and pyrimidine metabolism	methylphosphate	GC/MS	37070			0.1963	0.3040
1042	Cofactors and vitamins	Ascorbate and aldarate metabolism	threonate	GC/MS	27738	C01620	HMDB00943	0.4325	0.8531
1055		Hemoglobin and porphyrin	heme*	LC/MS pos	32593	C00032	HMDB03178	0.9972	0.5354
1056		Hemoglobin and porphyrin metabolism	bilirubin (Z,Z)	LC/MS neg	27716	C00486	HMDB00054	0.1036	0.8803
1057			bilirubin (E,E)*	LC/MS pos	32586			0.0002	0.5194
1059			biliverdin	LC/MS neg	2137	C00500	HMDB01008	0.4405	0.4311
1063		Nicotinate and nicotinamide metabolism	nicotinamide	LC/MS pos	594	C00153	HMDB01406	0.5907	0.9073
1084			trigonelline (N'-methylnicotinate)	LC/MS pos	32401	C01004	HMDB00875	0.4790	0.7500
1085		Pantothenate and CoA metabolism	pantothenate	LC/MS pos	1508	C00864	HMDB00210	0.3307	0.6305
1105		Tocopherol metabolism	alpha-tocopherol	GC/MS	1561	C02477	HMDB01893	0.9622	0.6682
1106			beta-tocopherol	GC/MS	35702	C14152	HMDB06335	0.8902	0.7042
1108			gamma-tocopherol	GC/MS	33420	C02483	HMDB01492	0.2469	0.0848
1117		Vitamin B6 metabolism	pyridoxate	LC/MS neg	31555	C00847	HMDB00017	0.8019	0.3829
1123		Benzoate metabolism	hippurate	LC/MS neg	15753	C01586	HMDB00714	0.3676	0.9387
1127			4-hydroxyhippurate	LC/MS neg	35527			0.2969	0.7926
1134			catechol sulfate	LC/MS neg	35320	C00090		0.4287	0.4776
1135			benzoate	GC/MS	15778	C00180	HMDB01870	0.1790	0.7350
1147			4-ethylphenylsulfate	LC/MS neg	36099	C13637		0.9064	0.4160
1148			4-vinylphenol sulfate	LC/MS neg	36098	C05627	HMDB04072	0.0002	0.3965
1151		Chemical	glycolate (hydroxyacetate)	GC/MS	15737	C00160	HMDB00115	0.7324	0.9966
1152			iminodiacetate (IDA)	GC/MS	21025		HMDB11753	0.5378	0.2340
1157			glycerol 2-phosphate	GC/MS	27728	C02979,D01488	HMDB02520	0.1901	0.5172
1191.5			Isobar: 2-propylpentanoic acid, 2-ethylhexanoic acid	LC/MS neg	35490			0.8846	0.5770
1212			salicylate	LC/MS neg	1515	C00805	HMDB01895	0.9799	0.3249
1216			4-acetaminophen sulfate	LC/MS neg	37475	C06804	HMDB01859	0.7846	0.8057

1217	Xenobiotics	Drug	4-acetamidophenol	GC/MS	12032	C06804	HMDB01859	0.7846	0.8057
1218			p-acetamidophenylglucuronide	LC/MS neg	33423		HMDB10316	0.7846	0.8058
1219			2-hydroxyacetaminophen sulfate*	LC/MS neg	33173			0.7846	0.8057
1220			2-methoxyacetaminophen sulfate*	LC/MS neg	33178			0.7846	0.8057
1221			3-(cystein-S-yl)acetaminophen*	LC/MS pos	34365			0.7846	0.8058
1222			2-methoxyacetaminophen glucuronide*	LC/MS pos	33161			0.7846	0.8058
1265.3			citalopram	LC/MS pos	38651	C07572	HMDB05038	0.8596	0.9915
1268.9			olanzapine	LC/MS pos	35971	C07322	HMDB05012	0.3647	0.3647
1269		EDTA	EDTA	LC/MS neg	32511	C00284		0.1903	0.8665
1272		Food component/Plant	tartarate	GC/MS	15336	C00898	HMDB00956	0.7028	0.4926
1286			quinate	GC/MS	18335	C00296	HMDB03072	0.6853	0.2794
1301			piperine	LC/MS pos	33935	C03882		0.6784	0.8327
1328			thymol sulfate	LC/MS neg	36095	C09908	HMDB01878	0.7195	0.0135
1341.04			stachydrine	LC/MS pos	34384	C10172	HMDB04827	0.2951	0.7620
1341.05			homostachydrine*	LC/MS pos	33009	C08283	HMDB04827	0.4564	0.0962
1343		Xanthine metabolism	caffeine	LC/MS pos	569	C07481	HMDB01847	0.5617	0.7568
1344			paraxanthine	LC/MS pos	18254	C13747	HMDB01860	0.7181	0.0771
1345			theobromine	LC/MS pos	18392	C07480	HMDB02825	0.4912	0.6404
1346			theophylline	LC/MS neg	18394	C07130	HMDB01889	0.5548	0.2235
1349			1,7-dimethylurate	LC/MS neg	34400	C16356	HMDB11103	0.2629	0.3429
1353			3-methylxanthine	LC/MS pos	32445	C16357	HMDB01886	0.8913	0.2391
1354			7-methylxanthine	LC/MS pos	34390	C16353	HMDB01991	0.9423	0.2887
1360		Sugar, sugar substitute, starch	erythritol	GC/MS	20699	C00503	HMDB02994	0.5261	0.5325
